# Predictive values of an immunological fecal occult blood test for the diagnosis of colorectal cancer compared using colonoscopy in symptomatic patients in Yaounde (Cameroon)

**DOI:** 10.1186/s12876-024-03292-x

**Published:** 2024-06-14

**Authors:** Tchuitcheu Ulrich Jovanka, Ndjitoyap Ndam Antonin Wilson, Bekolo Nga Winnie, Ngogang Marie Paule, Talla Paul, Dang Babagna Isabelle, Biwole Sida Magloire

**Affiliations:** 1 Department of Internal Medicine, Higher Institute of Medical Technology, University of Douala, Yaoundé, Cameroon; 2 Department of Internal Medicine, Faculty of Medicine and Biomedical Sciences, University of Yaoundé 1, Yaoundé, Cameroon; 3 Hepatogastroenterology Unit, Internal Medicine Service, Yaoundé General Hospital, Yaoundé, Cameroon; 4 Department of Clinical Sciences, Faculty of Medicine and Pharmaceutical Sciences, University of Douala, Douala, Cameroon

**Keywords:** Predictive value, Immunological fecal occult blood test, Diagnosis, Colorectal cancer, Symptomatic patients, Cameroon

## Abstract

**Introduction:**

The predictive value of immunological fecal occult blood (iFOB) testing for the screening of colorectal cancer has been well described in the Western world. However, its relevance in Sub-Saharan Africa (SSA) is not well evaluated. It could be altered by the other causes of lower gastrointestinal bleeding such as parasitic infections. The aim of this study was to highlight the performance of an iFOB test for the prediction of colorectal cancer (CRC) during colonoscopy in SSA.

**Methodology:**

We conducted an analytical cross-sectional study in two digestive endoscopic centers of Yaoundé (Cameroon) from the 1^st^ July to the 31 November 2022. Patients presenting with an indication for colonoscopy without any overt gastrointestinal bleeding were included. Sociodemographic and clinical data were collected. All consenting patients underwent a qualitative immunologic occult test through the iFOB test before colonoscopy. Data were analyzed using SPSS version 23.0 software. The performance of the iFOB test for the diagnosis of CRC during colonoscopy was evaluated in terms of sensitivity (Se), specificity (Sp), positive predictive value (PPV) and negative predictive value (NPV).

**Results:**

We included 103 patients during the study period with a male predominance and a sex ratio of 1.7. The median age [IQR] was 52 [38—65] years (range 1 – 84 years). The most common colonoscopic lesions were polyps in 23 patients (22.3%), CRC in 17 patients (16.5%) and hemorrhoids in 15 patients (14.6%). Patients testing positive for iFOB test accounted for 43.7% (45 patients). Among these patients, 31.1% (14 patients) had a CRC. The Se of the occult blood test for CRC detection was calculated to be 82.3% (95%CI: 56.7—96.2); the Sp was 63.9% (95% CI: 53—74); the PPV was 31.1% (95% CI: 24—39) and the NPV was 94.8% (95% CI: 86.6—98.1).

**Conclusion:**

The iFOB test has a good NPV, but a poor PPV for the diagnosis of CRC in our study.

## Introduction

The Colorectal cancer (CRC) is a major public health problem worldwide. According to the World Health Organization (WHO), the CRC is the 3th most common cancer in the world with an incidence of one million of new cases per year and 550,000 deaths [1]. In developed countries, CRC is the 2nd cause of cancers with an incidence of 520,000 new cases per year [1]. This high incidence has led to screening policies in developed countries and thus enabled early diagnosis and improvement in prognosis. It concerns asymptomatic people aged 45–75 years [1]. Several organizations, including the American Society of Clinical Oncology, have published resource-stratified guidelines to guide the implementation of CRC screening and early detection policy [2]. This led to a decrease in the incidence and the mortality due to CRC in the United States since 1985 [1, 3]. Improving the prognosis of CRC therefore requires early detection of precancerous lesions such as polyps and of cancer itself in its early stages [4]. Many tests are available for screening: fecal occult blood testing, flexible sigmoidoscopy, colonoscopy, or double-contrast barium enema [5].

The prevalence of CRC is increasing in sub-Saharan African (SSA) countries [6]. There is a lack of screening policies leading to poor prognosis [7]. In Cameroon, mortality is high due to late diagnosis at an advanced stage 4 in 60% of patients coupled with insufficiency in treatment [6, 8].

In SSA, the population is poor, with a life expectancy of 50–60 years. The age of onset of CRC seems to be younger with an average age of 50 years old, with many patients aged less than 30 years [8, 9]. The screening policy cannot be the same with those in developed countries [6]. We need to propose alternative diagnostic methods which are cheap, less invasive, and efficient.

The main objective of CRC screening is early detection to reduce mortality [10]. Several screening methods are available, including a total colonoscopy, which is the gold standard for CRC diagnosis [4]. But this is expensive and invasive with some risks such as a perforation [3, 11]. For this reason, some non-invasive tests have been developed and their predictive value with regards to colonoscopy are evaluated. Among these tests, occult blood testing is still the most widely used test because of its simplicity, accessibility and cost [12]. The guaiac test reacts to the peroxidase activity of heme, but this makes the test liable to reaction with other peroxidases in the feces, such as those from certain fruits, vegetables, and red meat. Dietary restrictions are therefore necessary to avoid false-positive results and poor compliance [1]. The immunologic tests seems to have a higher accuracy, sensitivity and specificity than the guaiac fecal occult blood testing [2]. Designed to specifically detect low levels of fecal occult blood, hHB ≥ 50 ng/mL without dietary restriction. They are clear, easy-to-interpret result. It is useful to detect bleeding caused by a number of gastrointestinal disorders, such as diverticulitis, colitis, polyps, and colorectal cancer [10]. Sometimes, the test could be self-administered by the patient [13]. Fecal occult blood tests are recommended for use in routine physical examinations, routine hospital testing, screening for colorectal cancer or gastrointestinal bleeding from any source [2]. Though this test has been validated in developed countries, its relevance for the diagnosis of CRC in SSA should be evaluated [2]. The socioeconomic development (such as the implementation of health insurance to promote screening guidelines) are different in these two areas [2]. Furthermore, the high prevalence of parasitic infections such as schistosomiasis could alter the efficacy of the immunological fecal occult blood (iFOB) test [2, 14]. Negative results do not exclude bleeding since it can be intermittent. False negative results may occur when occult blood is not evenly distributed throughout the bowel movement and fecal formation [15]. Despite its advantages, the relevance of the use of iFOB test for the diagnosis of CRC have been rarely evaluated in SSA [12].

### Objective

The objective of this work is to highlight the predictive value of an iFOB test for the diagnosis of a CRC in symptomatic patients during colonoscopy in the city of Yaoundé (Cameroon).

## Materials and method

### Type, duration and population of study

We conducted a cross-sectional and prospective study in the city of Yaoundé (Cameroon). The study sites were the endoscopic units of the Centre Médical la Cathedrale (CMC) and the Yaoundé General Hospital (YGH). The study was conducted over a period of 5 months from the 1st July to the 31 November 2022. We included all patients received at the digestive endoscopic unit with the following indications for examination: abdominal pain, change in bowel habits (chronic diarrhea, chronic constipation, alternating diarrhea/constipation), chronic anemia without evidence of bleeding, abdominal mass, rectal syndrome, whether associated with an alteration in general state or not. Patients with overt bleeding in stools declared by the patient or observed during stool collection were excluded. These patients underwent qualitative immunological testing for occult blood before colonoscopy.

### Variables studied

The variables studied were socio-demographic (age, gender), clinical (alcohol and tobacco consumption, indications for endoscopy) and paraclinical (occult blood test result and endoscopic results with anatomopathologic exam).

### The occult blood test procedure

**Fig. 1:**
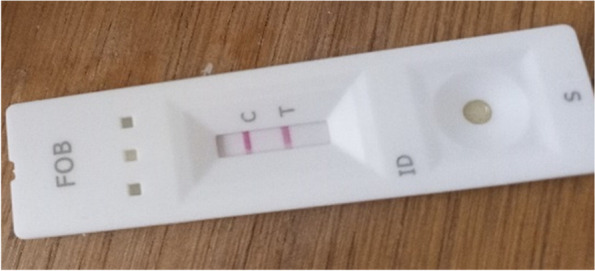
A positive iFOB test

**Fig. 2:**
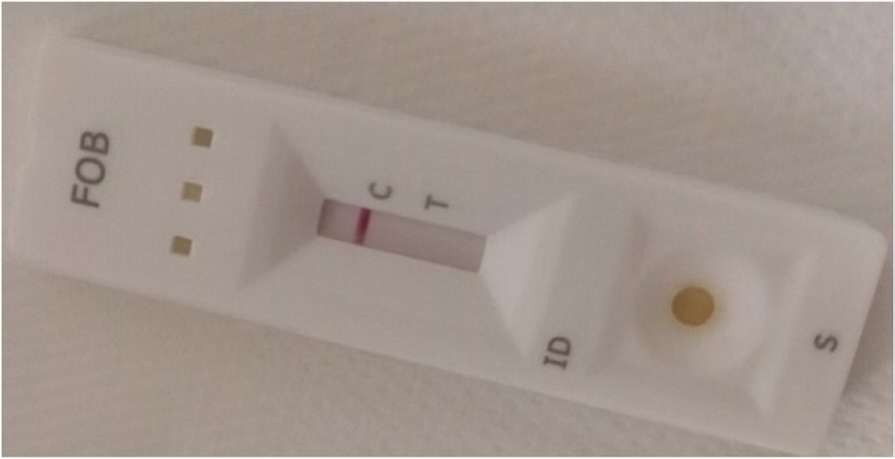
A negative iFOB test

The FOB (Fecal Occult Blood) test is an in-vitro immuno-chromatographic assay for qualitative detection of human hemoglobin in feces [15]. This exploration was realized with the “On site®” iFOB rapid test CE of the CTK Biotech laboratory, Poway, United States of America. For CRC detection, it has a relative sensitivity of 95.8% and a relative specificity of 98.8% in comparison with the reference test. Based on laboratory recommendations, we proceeded as follows:

1. *Collection of fecal samples:* in the toilet, a stool sample was collected in a clean, dry bottle by the patient.
2. *Processing of fecal samples (in the laboratory):* the examiner opened the stool collection device by unscrewing the top and used the collection stick to randomly prick the stool sample at five different sites. The collection stick was re-inserted and the bottle was sealed. The stool sampler was shaken vigorously to extract human hemoglobin from the sample.
3. *Test procedure:* The pocket was opened at the notch and the cassette removed. The stool collection device was shaken vigorously to ensure a homogeneous liquid suspension. Two drops were placed in the sample well of the cassette. The results were read after 5–10 min.
4. *Result:* The result could be positive, negative or invalid based on the recommendations of the fabricant. In the result window, a positive test had the presence of a test line and a control line (Fig. 1). A negative test had only a control line (Fig. 2). And an invalid test neither had a test line nor a control line [15].

### Colonoscopy and anatomopathologic examination

Endoscopic explorations were realized by 4 gastroenterologist doctors competent in digestive endoscopy. After three days of a special dietary regimen, the patient drank an osmotic laxative made of polyethylene glycol to empty the bowel. Then colonoscopy was realized with a Video endoscope Storz® at the Yaoundé General Hospital and with a Video endoscope Fujinon® at the Centre Medical la Cathédrale. Endoscopists were blinded to the iFOB test results. During the exam, the patient received a sedation based on Benzodiazepine drugs associated with an antispasmodic medication. Data collected from colonoscopy included the quality of preparation, the type and site of lesions, biopsy realization. In case of a suspicious tumoral lesion, the histopathological analysis was available within 10 days after colonoscopy. This last specifies the benign (polyp) or malignant (CRC) characteristic of the lesion.

### Data collection and analysis

Data were collected using a collection sheet, recorded using CSPRO 7.7 software and analyzed using SPSS version 23.0 software. The performance of the immunoassay in terms of sensitivity (Se), specificity (Sp), positive predictive value (PPV) and negative predictive value (NPV), was calculated for CRC diagnosis at colonoscopy with the 95% CI.

## Results

Over 253 patients were seen for colonoscopy in these digestive endoscopic units, 150 patients were excluded due to the presence of overt bleeding in their stools. Thus, we enrolled 103 patients who were tested for occult blood before realization of the colonoscopy.

The median age [IQR] was 52 [38- 65] years (range 1—84). We included 65 males (63.1%) and 38 females (36.9%) for a sex ratio of 1.7 (Table 1).

**Table 1:** Characteristics of the study population

Variable	Number (*n* = 103)	Percentage
**Median age**	52 ± 13 years (range 1—84)	
**Sex**		
-Male	65	63.1%
-Female	38	36.9%
***Associated risk factors***		
-Alcohol consumption	66	64.1%
-Tobacco consumption	9	8.7%
***Indications of the colonoscopy***		
-Abdominal pain	79	76.7%
-Constipation	55	53.4%
-Altered general status	26	25.2%
-Diarrhea	16	15.5%
-Alternating diarrhea-constipation	8	7.8%
-Rectal syndrome	5	4.8%
-Abdominal mass sensation	4	3.8%
-Anemia	3	2.9%
***Lesions observed after the colonoscopy***		
-Polyps	23	22.3%
-Colorectal cancer	17	16.5%
-Hemorrhoids	15	14.6%
-Diverticular disease	6	5.8%
-Chronic inflammatory bowel disease	2	1.9%
-Other lesions	8	7.8%

### Associated risk factors

We observed 66 patients (64.1%) with alcohol consumption and 9 patients (8.7%) with tobacco consumption (Table 1).

### Indications for colonoscopy

Abdominal pain was the main indication for colonoscopy in 79 patients (76.7%). It was followed by constipation with 55 patients (53.4%), altered general status with 26 patients (25.2%), diarrhea in 16 patients (15.5%), alternating diarrhea-constipation in 8 patients (7.8%), rectal syndrome in 5 patients (4.8%), abdominal mass sensation in 4 patients (3.8%) and anemia in 3 patients (2.9%) (Table 1). We noted that a patient could have more than one indication of colonoscopy.

### Lesions observed during colonoscopy

In total, 103 patients were registered, 60 (58.3%) had at least one colorectal lesion and the exam was normal in 43 patients (41.7%). There were polyps in 23 patients (22.3%), CRC in 17 patients (16.5%), hemorrhoids in 15 patients (14.6%), diverticular disease in 6 patients (5.8%), chronic inflammatory bowel disease in 2 patients (1.9%), and other lesions 8 patients (7.8%) (Table 1).

### The iFOB test performance

Of the 103 patients included, 45 (43.7%) had a positive occult blood test and 58 a negative test.

**Fig. 3:**
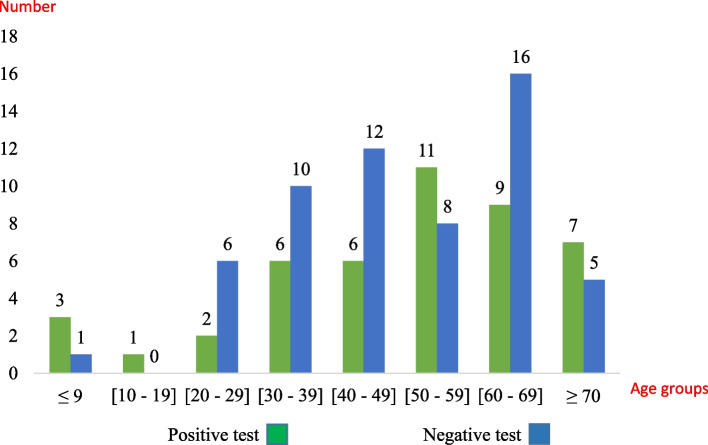
Repartition of iFOB test results regarding the age class

A positive test could be observed in any age group (Fig. 3).

Over 17 patients with a CRC lesion, 14 had a positive occult blood test and 3 a negative test (Table 2). Sensitivity was calculated to be 82.4% (95% CI 56.7—96.2).

**Table 2:** Distribution of colorectal cancer lesion according to occult blood test result

Variables	Colorectal cancer		Total
	**Yes**	**No**	
**Occult blood test result**			
Positive	14 (13.6%)	31 (30.1%)	45 (43.7%)
Negative	3 (2.9%)	55 (53.4%)	58 (56.3%)
**Total**	17 (16.5%)	86 (83.5%)	103 (100%)

Of the 86 patients without a CRC lesion, 55 had a negative occult blood test and 31 a positive test (Table 2). This gave us a specificity of 63.95% (95% CI 53—74).

In the subgroup of negative tested patients, 3 had CRC and 55 did not (Table 2). This allowed us to calculate the positive and negative predictive value which were 31.1% (95% CI 24—39) and 94.8% (95% CI 86.6—98.1) respectively.

## Discussion

Concerning the sample size, we excluded 150 patients (59.3%) of them due to the presence of overt blood in their stools. This is consistent with other studies which have described lower digestive bleeding as a main indication of colonoscopy [11, 16]. The iFOB test is indicated in patients without any visible blood in their stool.

We obtained a median age [IQR] for CRC of 52 [38- 65] years years. This result is similar to that obtained by Katilé et al. in Mali in 2021, where the mean age was 48.6 years with extremes ranging from 15 to 81 years [16]. In Nigeria, Knapp et al. conducted a study, where the median age was 51 years (46—58) [2]. These median age are younger than those observed in western studies [1]. Given that the life expectancy in SSA country is low, the screening policy should be earlier than in western countries [6]. Some CRC are diagnosed before 30 years [9].

In this study, a male predominance with a sex ratio of 1.7 was highlighted. Ankouane et al., in a study conducted in Cameroon in 2013 also noted a male predominance [11]. WHO also describe a male predominance [1].

In our series, the most common indications were: abdominal pain (76.7%), constipation (53.4%), followed by altered general status (25.2%). These results are different from those obtained in Cameroon in 2013 by Ankouane et al., and those highlighted by Katilé et al. in 2021 in Mali, all of whom found rectal bleeding as the first indication, followed by abdominal pain and finally transit disorders [11, 16]. This difference is attributed to the exclusion of rectal bleeding in our study. This concerned 150 patients, of the 253 who did a colonoscopy within the study period.

This study found a significant number of patients for which colonoscopy indication was a change in bowel habits. Most of them was constipation, but we also found diarrhea or alternation between diarrhea and constipation. El Housse et al., in Morocco in 2015, identified a symptomatology dominated by transit disorders, diarrhea and/or constipation in a study of the anatomoclinical profile of patients with CRC [17]. A large mass of CRC could lead to a large bowel obstruction responsible for these symptoms.

We obtained a positive occult blood test in 45 patients (43.7%). This result is higher than rates observed in Lagos urban area and other regions in Nigeria. The Lancet review "Colorectal cancer screening in sub-Saharan Africa" reveals the positivity rates of 11% in Lagos, 20% in Osun, and 28% in Kwara [6]. The high frequency of positivity of the occult blood test in our sample could be explained by the criteria’s selection of patients. In our study, we included symptomatic patients with symptoms such as abdominal pains, constipation, diarrhea, and bloating. There are some other digestive disorders justifying the need for colonoscopy without any macroscopic visible blood in their stools. But these symptoms could also suggest a colonic lesion, increasing the risk of microscopic bleeding. While the iFOB test has been developed for asymptomatic patients without macroscopic digestive bleeding nor other signs [6], the inclusion of symptomatic patients could explain the high prevalence of positive test in our study.

Concerning the diagnostic value of the iFOB test, we observed a poor PPV estimated to only 31.1% to predict a CRC on colonoscopy and a histopathological examination. A positive iFOB test could be a CRC but also a diverticular disease, a polyp, a chronic inflammatory bowel disease or other. This poor PPV in SSA countries have also been described in Nigeria by Knapp et al. in 2021 and by Alatise et al. in 2022 [2, 10]. For this reason, any patient who is able to do a colonoscopy should benefit from this endoscopic exploration if possible. Colonoscopy remains the gold standard for colonic exploration.

On the other hand, with an NPV of 94.8%, the iFOB test appears as a good tool to identify patients without any lesion on colonoscopy. Thus, in settings where access to digestive endoscopy is limited, such as in SSA countries, it can be used to select patients for whom it is less useful to realize colonoscopy. With these results, we can suggest that for patients presenting with abdominal pain, constipation or diarrhea, altered general status, anemia with a negative iFOB test, the risk of having CRC is low. But we cannot assert that these patients should be exempted from colonoscopy. First, all patients with CRC do not have positive iFOB; in our study, 3(17%) out of 17 were negative. Secondly, these symptoms could be due to another significative lesion such as diverticular disease, irritable bowel disease or colitis. The iFOB test has been developed for the screening of the CRC in asymptomatic people [1]. But looking at the specific context of SSA, a screening program should be adapted [6].

With a Se of 82.3% (CI: 56.7—96.2) and a Sp of 63.9% (CI: 53—74), the predictive value of the iFOB test for CRC diagnosis seem less efficient than laboratory estimation. This difference could be explained by the relative young age of patients in our study. In western countries, the IFOB test is indicated in any patient aged from 50 to 75 years old [1]. Those with a positive test benefit of a screening colonoscopy looking for a CRC. In our study, we did the test in patients from any age who already had an indication of colonoscopy. Thus the average age of our population was lower. With this relatively young age, the incidence rate of CRC was lower than in western studies. We observed a normal exam in 43 patients (41.7%), polyps in 23 patients (22.3%), CRC in 17 patients (16.5%), hemorrhoids in 15 patients (14.6%), diverticular disease in 6 patients (5.8%), chronic inflammatory bowel disease in 2 patients (1.9%), and other lesions 8 patients (7.8%).

### Limitation

The main limitation of our study is the small size of the sample. Moreover, we did not look for the predictive value of the iFOB to diagnose pre-cancerous lesions such as polyps.

### Added value of this study

Our study confirms the good diagnostic performance of an iFOB test with regard to colonoscopy in a SSA despite some confounding factors. This test could be used to select patients who could be exempted from an endoscopic exploration for CRC screening.

## Conclusion

The iFOB test has a good NPV for the diagnosis of CRC in our study. Looking at the good NPV, it can allow for selection of patients who do not need a diagnostic colonoscopy for CRC in low income areas. On the other hand, the PPV value is poor, limiting the expansion of this test for the screening in our country.

## Data Availability

Authors confirm that all data and materials are available. The investigator Tchuitcheu Ulrich Jovanka should be contacted at jovankatchuitcheu8@gmail.com, if someone wants to request the data from this study.
